# Genome-wide miRNA analysis and integrated network for flavonoid biosynthesis in *Osmanthus fragrans*

**DOI:** 10.1186/s12864-021-07439-y

**Published:** 2021-02-27

**Authors:** Yong Shi, Heng Xia, Xiaoting Cheng, Libin Zhang

**Affiliations:** 1 College of Life Science and Technology, Huazhong University of Science and Technology, Wuhan, 430074 China; 2 Department of Bioinformatics and Systems Biology, Hubei Bioinformatics & Molecular Imaging Key Laboratory, College of Life Science and Technology, Huazhong University of Science and Technology, Wuhan, 430074 China

**Keywords:** *Osmanthus fragrans*, MicroRNAs, Deep sequencing, qRT-PCR, Target genes

## Abstract

**Background:**

*Osmanthus fragrans* is an important economical plant containing multiple secondary metabolites including flavonoids and anthocyanins. During the past years, the roles of miRNAs in regulating the biosynthesis of secondary metabolites in plants have been widely investigated. However, few studies on miRNA expression profiles and the potential roles in regulating flavonoid biosynthesis have been reported in *O. fragrans.*

**Results:**

In this study, we used high-throughput sequencing technology to analyze the expression profiles of miRNAs in leaf and flower tissues of *O. fragrans*. As a result, 106 conserved miRNAs distributed in 47 families and 88 novel miRNAs were identified. Further analysis showed there were 133 miRNAs differentially expressed in leaves and flowers. Additionally, the potential target genes of miRNAs as well as the related metabolic pathways were predicted. In the end, flavonoid content was measured in flower and leaf tissues and potential role of miR858 in regulating flavonoid synthesis was illustrated in *O. fragrans*.

**Conclusions:**

This study not only provided the genome-wide miRNA profiles in the flower and leaf tissue of *O. fragrans*, but also investigated the potential regulatory role of miR858a in flavonoid synthesis in *O. fragrans*. The results specifically indicated the connection of miRNAs to the regulation of secondary metabolite biosynthesis in non-model economical plant.

**Supplementary Information:**

The online version contains supplementary material available at 10.1186/s12864-021-07439-y.

## Background

MicroRNAs (miRNA) are a class of non-coding single-stranded RNA molecules with length about 21 nucleotides encoded by endogenous genes. In animals and plants, miRNAs post-transcriptionally regulate gene expression through either the mediation of target mRNAs degradation or the inhibition of target mRNAs translation. It is well known that miRNAs binds to RNA-induced silencing complex (RISC), where the target mRNA degradation is catalyzed [1, 2]. To target mRNA for degradation, miRNAs and their target genes are nearly perfectly complementary pairing [3]. In former studies from other groups, the roles of miRNAs in plant development have been well illustrated. For instance, miRNAs participate in the regulation of numerous biological processes, such as cell proliferation, leaf and root development, phase transition [4–6].

Most miRNAs are conserved during evolution and can be identified by traditional sequence homology analysis [7]. However, some miRNAs are specifically expressed in certain plant species at comparatively low levels, which makes the identification difficult by traditional experimental approaches [8, 9]. Due to the emergence and development of deep sequencing technology, the species-specific or low-abundance miRNAs can be effectively detected, therefore accelerating the study of miRNAs function in diverse plant species, for instance, *Arabidopsis* [9], rice [10], tomato [11], *Zea mays* [12], *Brassica napus* [13], Chinese cabbage [14], and potato [15, 16]. In addition, the deep sequencing technology has been widely used to identify the miRNAs in non-model plant species. *O. fragrans* is one of the most known medicinal plants in China, typically used in folk medicine as expectorant and anti-cough agent for thousand years. It is usually served as an additive in food, tea and other beverages [17, 18], and the flower oil of *O. fragrans* has a mildly sedative effect on controlling the energy balance of the body in terms of the prevention of over-eating and gaining weight [18–20]. Though many studies about *O. fragrans* development and application have been reported in the recent years [18, 19, 21–26], there is no study reported to address the genome-wide identification of the microRNAs in *O. fragrans,* which impeded the comprehensive understanding of the regulation networks during *O. fragrans* development. Therefore, entirely identifying the miRNAs and analyzing their functions in *O. fragrans* can further provide important additional information about the regulatory mechanisms in the biological processes of *O. fragrans* and will be useful to isolate high-quality and native medical or economical products from *O. fragrans*.

As secondary metabolites in plants, flavonoids have important regulatory roles in plant development [27, 28]. Including flavonoids, there are a large number of secondary metabolites were found to be accumulated in many tissues of *O. fragrans* [29, 30]. And interestingly, more and more evidence has shown that the biosynthesis and accumulation of secondary metabolite in plants were mediated by miRNAs [31–35]. For instance, miR156-targeted SPL9 was found to regulate the biosynthetic pathway of flavonoids [32], and the down-regulation of miR156 significantly induces the accumulation of flavonols [32]. In addition, miR858 was reported to putatively regulate MYB transcription factors in *A.thaliana* [34], and MYB family transcription factors MYB11, MYB12, as well as MYB111 were found to regulate flavonol biosynthesis by targeting CHI, CHS and F3H [36]. However, the regulation mechanism of flavonoids biosynthesis by miRNAs in *O. fragrans* has not been investigated yet.

In this study, the total RNA samples were isolated from *O. fragrans* leaf and flower tissue, and used to generate the small RNA libraries for sequencing analysis. After the measurement of deep sequencing by Illumina Hiseq 2000 platform, the sequence data quality was carefully checked. Via bioinformatical analysis, up to 107 conserved miRNAs and 88 novel miRNAs were identified from the sequencing data. Furthermore, to better understand the functions of the identified conserved and novel miRNAs, as well as the ways that they play regulatory functions in *O. fragrans*, GO analysis and KEGG analysis of the target genes were also performed. Our data suggested a potential regulatory role of miRNAs in flavonoids biosynthesis in *O. fragrans.*

## Results

### Sequence analysis of *O. fragrans* sRNA libraries

**Fig. 1:**
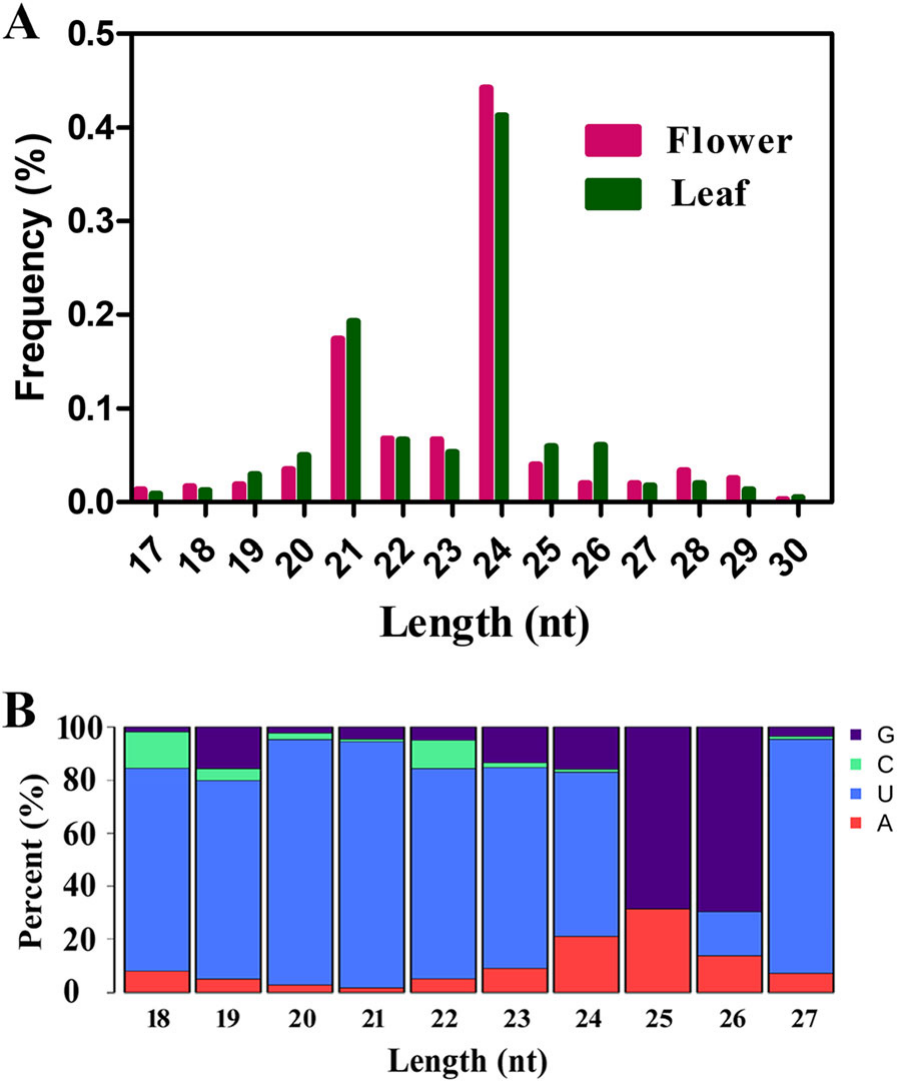
MiRNA sequencing analysis of flower and leaf tissues in *O. fragrans*. **a**. Length distribution and frequency analysis of miRNAs in *O. fragrans*. **b**. First nucleotide bias of miRNAs with different lengths. Y axis indicates the percent of first nucleotides in miRNAs and X axis indicates the miRNA length

To obtain the RNA sequencing data for identification of the conserved and novel miRNAs in *O. fragrans*, total RNA was firstly extracted from flower and leaf tissues and cDNA libraries were independently generated for sequencing measurement. To better study the miRNAs expression profile and functions, adaptor sequences and low-quality reads were carefully filtered. In the end, 22.83 and 23.13 million clean reads were acquired from flower and leaf groups (Additional file 1: Table S1), respectively. The lengths of small RNAs were distributed from 18 nt to 30 nt, with the majority in 20-24 nt, as shown in Fig. 1a. In the flower tissue of *O. fragrans*, 83.9% of small RNAs were 20-24 nt, while 81.2% in the tissue. Moreover, regardless of the samples source, most small RNAs were characterized with 24 nt in length, the percentages of 24 nt small RNAs were 44.2% in flowers and 41.3% in leaf. These results were in line with former studies of other plant species, such as *Arabidopsis* [9], rice [10], peanut [37] and *L. japonica* [38]. As shown in Additional file 1: Table S1, rRNA and tRNA represented the two most abundant small RNAs in both small RNA libraries. In the following data analysis, the rRNAs, tRNAs, snRNAs and snoRNAs were not included. To further address the tissue specificity of the small RNAs in *O. fragrans*, the comparative analysis of the small RNAs reads between flower and leaf tissue was performed. There were approximately 80% of clean reads detected in both tissues, and the remaining clean reads only detected in either flower or leaf.

### Conserved miRNA identification in *O. fragrans*

To identify the conserved miRNAs in *O. fragrans*, the obtained small RNAs sequences were compared with the identified miRNAs in other plants, which are available in miRBase database. Analysis result had shown there were 175 and 174 conserved miRNAs identified from the flower and leaf libraries, respectively (Additional file 2: Table S2). These conserved miRNAs can be further categorized into 47 families, among which 43 families were presented in both libraries. The read numbers of the conserved miRNAs in miRNA families varied dramatically from 1to 3,300,711 (Additional file 2: Table S2). Interestingly, miR-5538 family was specifically observed in flower tissue, while miR535 family was only identified in leaf tissue. It was reported that miR-535 was induced in leaves under low ambient temperature treatment [39], which is consistent with the analyzing result in this study since the sampling time was in October and the temperature was comparatively low in the middle areas of China. In addition, a total of four potential target genes of miR-5538 were found and one of them is TPIS_PETHY (P48495), which was annotated as triose-phosphate isomerase and plays a role in regulating corolla development [40]. To note, miR-166 family had the highest expression level in both flower and leaf tissue. In contrast, miR5538 family normally express at low level, but it exhibits important functions in regulating plant developmental processes including flower development and abiotic stress responses [14]. As mentioned before, TPIS_PETHY is one of the target genes of miR5538. It shares 84% sequence similarity with cytosolic tpi gene, which is finely regulated during flower development. Therefore, we speculate that over-expression of miR5538 will mediate corolla development [40]. Taken together, these results had shown that the expression levels of conserved miRNAs highly varied, which could be because of the tissue-specific or stage-specific expression patterns of conserved miRNAs in *O. fragrans*. However, since *O. fragrans* genome information is limited, it was difficult to further define the genomic loci for these conserved miRNA families.

### Novel miRNA identification in *O. fragrans*

To predict novel miRNAs in the obtained libraries, first of all, the hairpin structure of miRNA precursors is characterized. We performed the sequences folding analysis of potential miRNA precursors and identified the novel miRNAs in the libraries. To further confirm the candidate miRNAs, the Dicer cleavage sites as well as the minimum negative folding free energy were employed. In the end, 45 novel miRNAs in the flower library and 58 novel miRNAs in the leaf library were successfully detected (Additional file 3: Table S3). As shown in Additional file 3: Table S3, there were 15 novel miRNAs expressed in both tissues, while 73 novel miRNAs were tissue-specific expressed. Among the 73 tissue-specific novel miRNAs, 31 novel miRNAs were exclusively observed in flower and the other 42 novel miRNAs were in leaf. In the flower library, the lengths of mature novel miRNAs were from 18 to 24 nt, while the lengths ranged from 18 to 25 nt in the leaf library. Importantly, most miRNAs were 24 nt in length in both libraries, and the miRNAs with 21 nt were the second most abundant. Besides, the lengths of the novel miRNA precursors were also measured. It ranged from 59 to 226 nt in the flower library and from 58 to 226 nt in the leaf library, with the average lengths 92 nt and 87 nt, respectively. The MFEI of the precursor sequences ranges from − 0.67 to − 1.46 with an average of − 1.05 in flower and − 0.59 to − 1.80 with an average of − 1.03 in leaf, respectively. Furthermore, we performed miRNA bias analysis in *O. fragrans*. The results showed the miRNAs from flower and leaf tissues displayed similar nucleotide distribution pattern at first nucleotide position. For example, approximately 90% of nucleotides were U at first nucleotide in 21-nt miRNAs. In addition, approximately 60% nucleotides were U at the first nucleotide in 24 nt miRNAs (Fig. 1b). Taken together, these results indicated mature miRNAs had higher A–U content than G-C content at first nucleotide except for miRNAs with length 25 nucleotides and 26 nucleotides in *O. fragrans* (Fig. 1b).

Compared with the conserved miRNAs, the novel miRNAs in *O. fragrans* had much lower expression levels (Additional file 3: Table S3). In *O. fragrans*, ofr*-*Novel_35, ofr*-*Novel_46, ofr*-*Novel_62, ofr*-*Novel_64, ofr*-*Novel_67, ofr*-*Novel_84 and ofr*-*Novel_85 were most abundant in flower tissues, and the read numbers of novel miRNAs were normally less than 100. Meanwhile, eight novel miRNAs were most abundant in the leaf tissue, including ofr*-*Novel_14, ofr*-*Novel_46, ofr*-*Novel_64, ofr*-*Novel_67, ofr*-*Novel_75, ofr*-*Novel_79, ofr*-*Novel_84 and ofr*-*Novel_85. The majority (60.34%) of these novel miRNAs in leaf had less than 100 reads. Given the significant difference of the read numbers between the novel and conserved miRNAs in *O. fragrans*, these novel miRNAs may specifically express in certain tissues or at particular developmental stages. Moreover, the low-expression pattern of these novel miRNAs is consistent with former study for other important plant species [3].

### Differential expression analysis and target gene prediction of miRNAs in *O. fragrans*

**Fig. 2:**
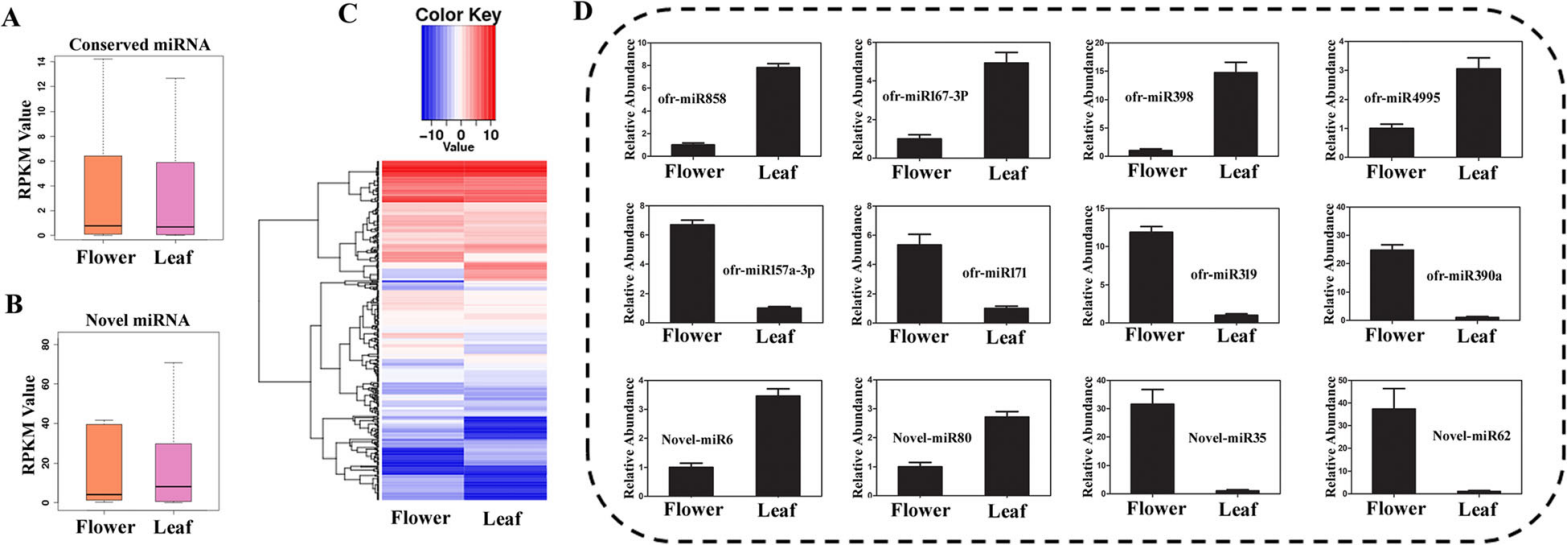
miRNA identification and differentially expressed miRNA analysis in *O. fragrans*. **a**. RPM distribution of conserved miRNA expression. **b**. RPM distribution of novel miRNA expression. **c**. Heatmap analysis of the differentially expressed miRNAs of flower and leaf tissues in *O. fragrans*. **d**. Validation of differentially-expressed miRNAs using qRT-PCR

MiRNAs play versatile roles in post-transcription regulation of the target genes expression, which are essential for plant development. By performing differential expression analysis using the DESeq software [41], there were 77 conserved miRNAs and 5 novel miRNAs up-regulated, as well as 63 conserved miRNAs and 7 novel miRNAs down-regulated in flower tissue, compared with that in leaf tissue (Fig. 2a, b, c; Additional file 4: Table S4). The reliability of the differentially expressed miRNAs was further confirmed by qRT-PCR experiments in which twelve randomly selected differentially expressed miRNAs were tested (Fig. 2d). To explore miRNA functions in *O. fragrans* development, we used the software psRobot to predict the potential target genes of miRNAs with the published criteria [42]. As a result, 2743 genes were identified as the potential targets of 159 conserved miRNAs, and 713 genes were predicted as the targets for 67 novel miRNAs (Additional file 5: Table S5). For different miRNAs, the number of potential target genes varied dramatically from 1 to 133, with the average number 15. Interestingly, some of the potential target genes of identified miRNAs belong to transcription factors, such as the well-known WRKY family protein and GRAS family protein. It is well known that GRAS gene family comprises several transcriptional regulators, and participates in the regulation of plant growth and development [43]. In addition, to validate the target gene of miRNA858a, miR858a cleavage site on its mRNA target MYB1 was detected by 5’RLM-RACE (Additional file 6: Fig. S1).

**Fig. 3:**
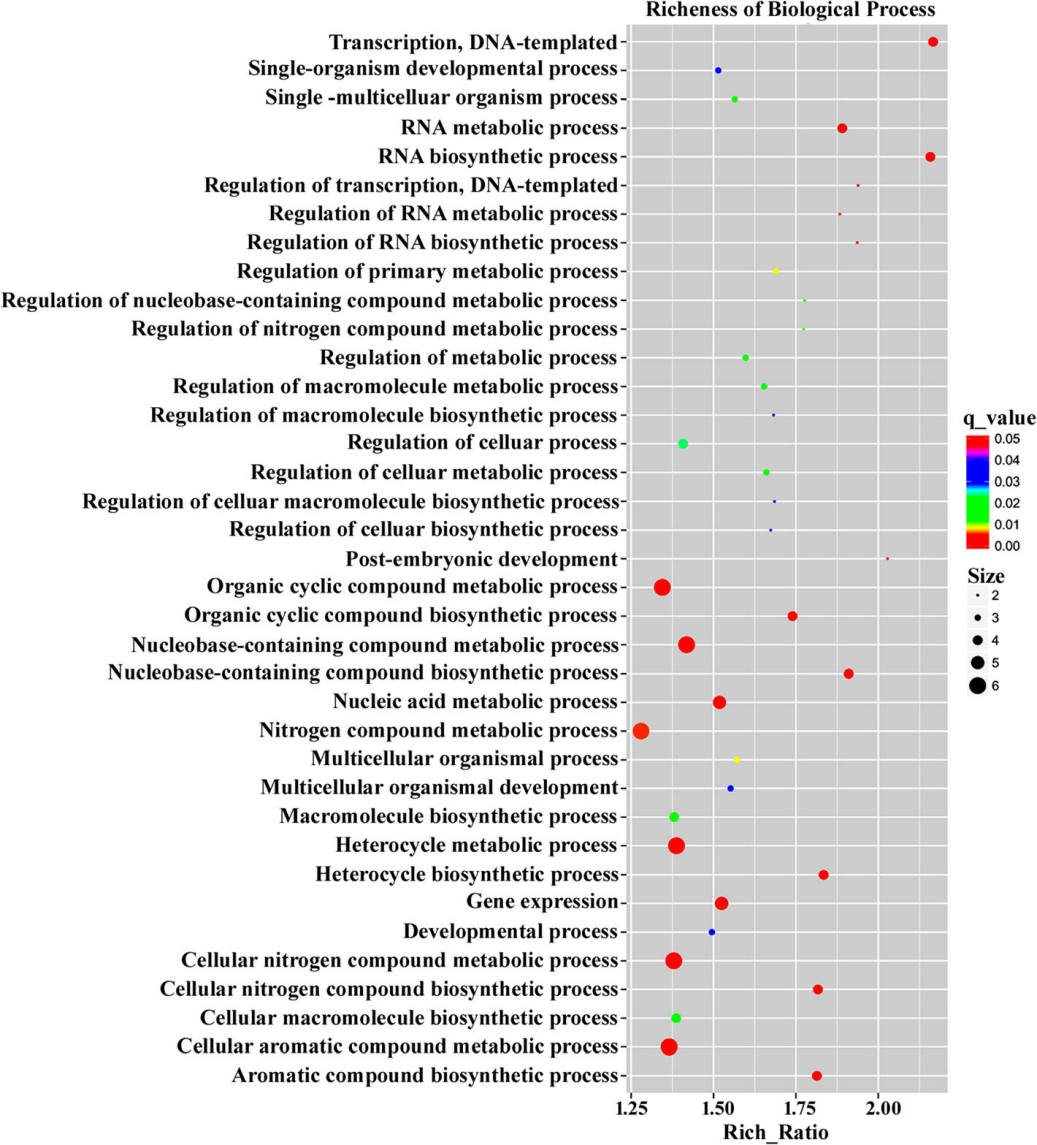
GO (Gene Ontology) analysis of the mRNA targets of differentially expressed miRNAs between flower and leaf tissues in *O. fragrans*

Afterwards, the potential targets were assigned with GO terms to address their biological functions. The involved GO terms were further categorized into 67 groups. To better determine the processes regulated by these target genes, we classified these 67 different groups into three main fields, cellular component (841 sequences), biological process (1361 sequences) and molecular function (1091 sequences). For example, in the biological process category, most GO terms were distributed in “organic cyclic compound metabolic process”, “organic cyclic compound biosynthetic process”, “nucleobase-containing compound metabolic process”, “nucleobase-containing compound biosynthetic process” and “heterocycle metabolic process” (Fig. 3). These results indicated the potential target genes of miRNAs participate in numerous cellular biosynthetic and metabolic processes, suggesting that the identified differentially expressed miRNAs play important roles in regulating cellular development and metabolic processes in *O. fragrans*.

To better understand the biological functions and underlying mechanisms of the potential target genes, we took advantage of the KEGG database to explore the biochemical pathways in which the target genes participate. As shown in Additional file 7: Table S6, there were 361 KEGG pathways obtained according to the target genes. Interestingly, several target genes of the novel miRNAs identified in *O. fragrans* can regulate spliceosome assembly (Top 1 pathway). Among them, some were the key members of spliceosome, such as U1, U2, U4, U5 and U6. Meanwhile, some other target genes directly affect the spliceosome assembly, including Prp5, Prp2, Prp16, Prp17, Prp18, Prp22, Slu7, Prp22 and Prp43. These results implied that the miRNAs identified in *O. fragran* may play regulatory roles through the modulation of alternative splicing, therefore mediated plant development and the responses to stresses. In addition, the KEGG pathways mainly localized in five different groups, cellular processes, environmental information processing, genetic information processing, as well as cell metabolism.

### Integrative network analysis of miRNA and target genes in *O. fragrans*

**Fig. 4:**
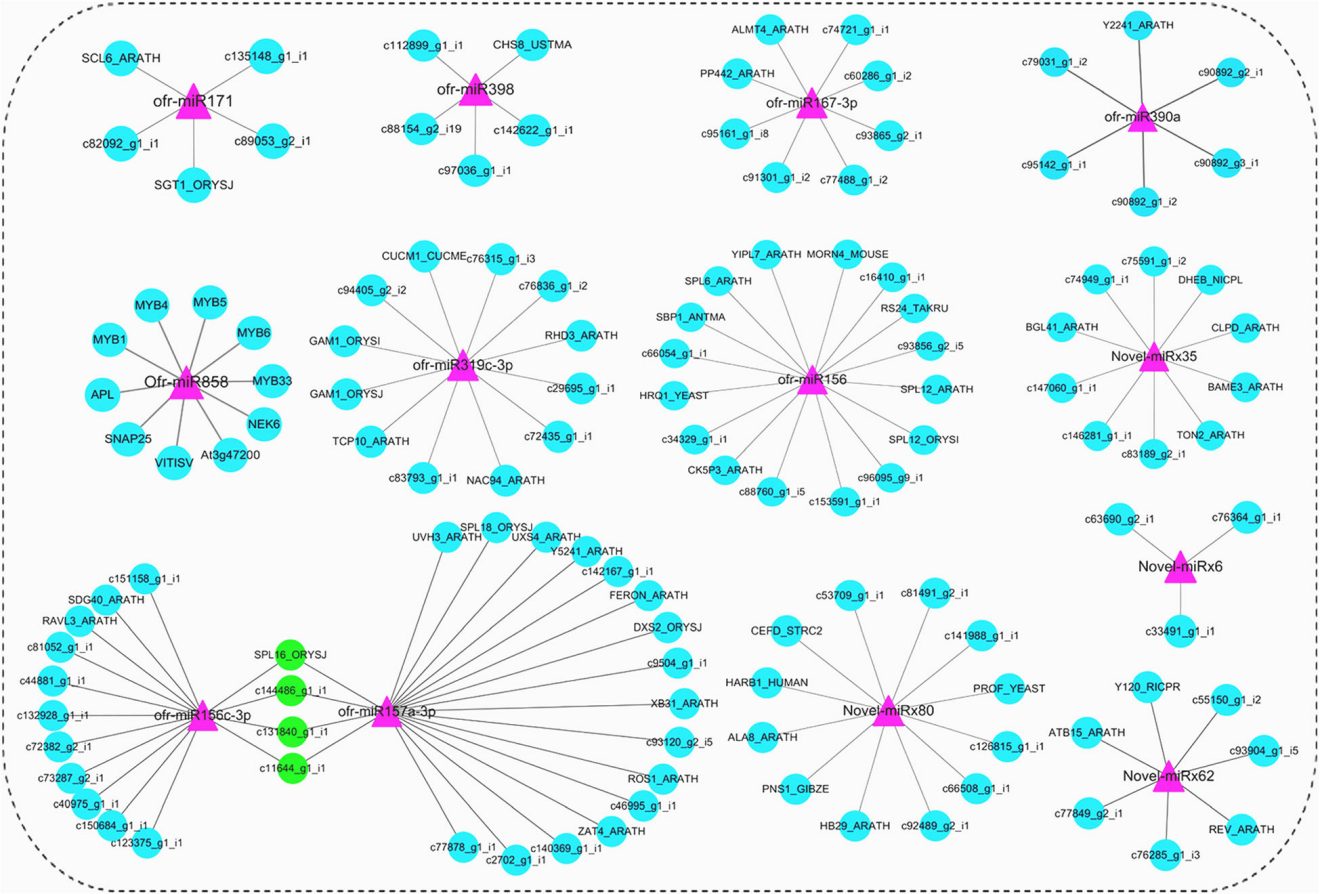
miRNA-mRNA interactive network in *O. fragrans* (miR171, miR398, miR167-3p, miR390a, miR 858, miR319c-3p, miR156, Novel-miRx35, miR156c-3p, miR167a-3p, Novel-miRx80, Novel-miRx6 and Novel-miRx62 were used as samples)

**Fig. 5:**
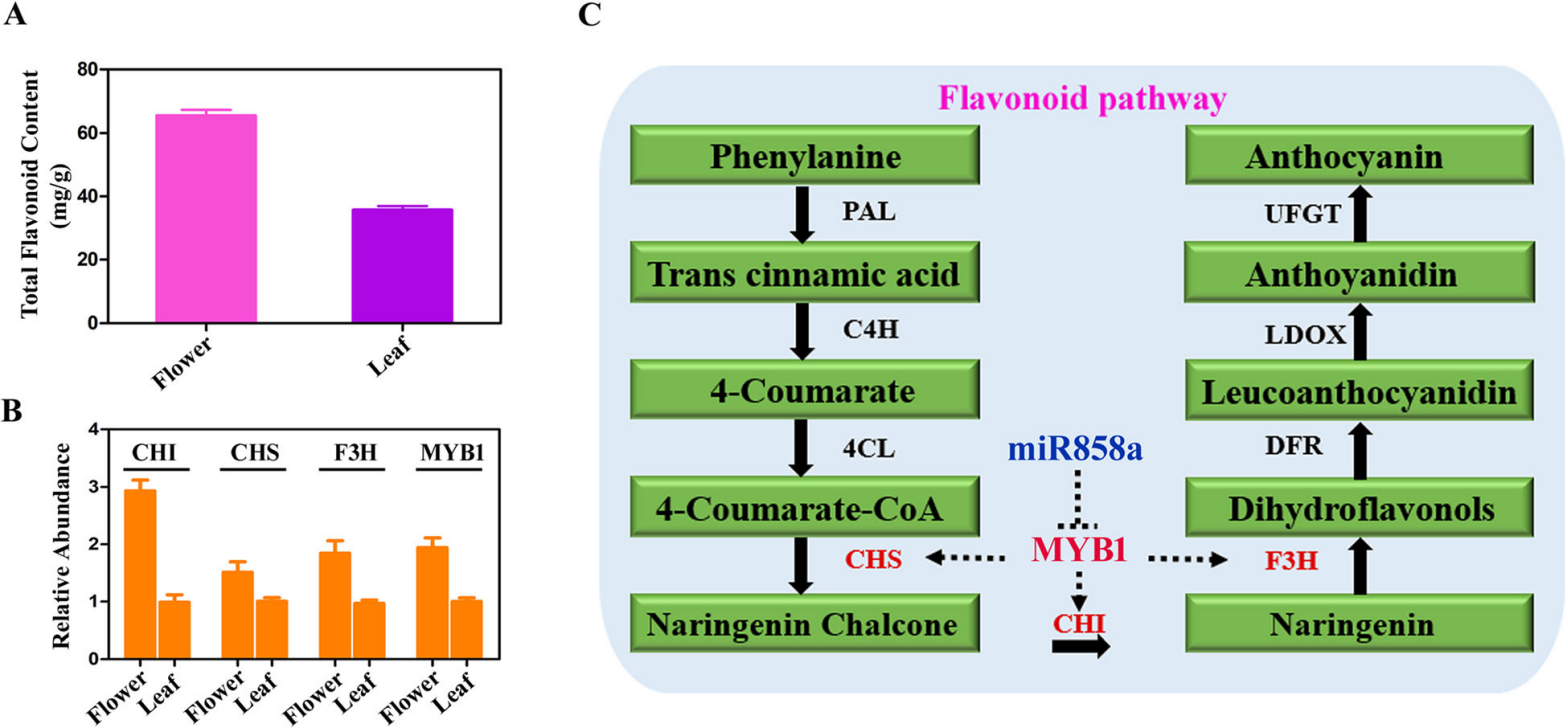
Analysis of flavonoid content and differentially expressed genes involved in flavonoid pathway. **a**. Flavonoid content analysis between flower and leaf tissues in *O. fragrans*. **b**. Analysis of differentially expressed genes involved in flavonoid pathway. **c**. The sketch map of speculative regulation pathway of flavonoid biosynthesis in *O. fragrans*

Integrative network analysis of miRNA and target genes was further performed in *O. fragrans*, which is useful to illustrate the biological functions of the miRNAs. In this study, there were 133 identified miRNAs differently expressed in flower and leaf tissues. These miRNAs were named as DE-miRNAs, including 88 novel miRNAs. In addition, the miRNA-mRNA networks of the DE-miRNAs were analyzed and some interesting mRNA targets were investigated, which were listed in Fig. 4. For instance, RHL41 has been reported to mediate the tolerance to high light and cold acclimation as a transcription factor, and it is the target of novel-miRx87 [44]. The flowers and leaves of *O. fragrans* were reported to contain several flavonoids, including rutin, isoquercitrin, quercitrin, and quercetin [29, 30]. Here, the flavonoid content was measured using a modified colorimetric method. As shown in Fig. 5a, flavonoid content in flower tissues of *O. fragrans* is appropriate 67 mg/g, which is significantly higher than that in leaf tissues (35 mg/g). Since the differential accumulation of flavonoids in flower and leave tissues was mainly regulated by the key genes in flavonoid metabolism pathway, we further investigated whether the identified miRNAs have effect on the functions of these genes in *O. fragrans*. Several miRNAs, including miR858, miR156 and miR172, were reported to play essential regulatory roles in flavonoid biosynthesis [45]. For example, miR858 was reported to putatively regulate MYB transcription factors in *A.thaliana* [34], while MYB transcription factors were found to regulate flavonol biosynthesis by interacting with CHI, CHS and FLS genes [35]. Importantly, our study also showed miR858a targets MYB genes in miRNA-mRNA network (Fig. 3). Moreover, we found miR858a was downregulated (Fig. 2) and MYB1 gene was upregulated in flower tissues of *O. fragrans* (Fig. 5b). Furthermore, our results showed that CHI, CHS and FLS genes were significantly up-regulated in flower tissues of *O. fragrans* (Fig. 5b). Taken together, these results suggested a negative correlation between miR858a level and MYB1 gene expression.

## Discussion

MiRNAs are important non-coding small RNA molecules which participate in the regulation of numerous physiological processes in plant [1]. Based on the advantages of high-throughput sequencing technology, the capacity in large-scale miRNAs detection and high sensitivity in the measurement of minimally expressed miRNAs, it has been widely used to powerfully identify conserved miRNAs and species-specific miRNAs during the past years. However, the comprehensive study of miRNAs detection in *O. fragrans*, one of the widely cultivated perennial, evergreen broad leaved trees in Asia, is not reported yet. In our study, small RNA libraries of flower and leaf tissues in *O. fragrans* were constructed using high-throughput sequencing technology, and about 22.83 million clean reads from flower, 23.13 million clean reads from the leaf library were obtained. Via bioinformatics analysis, there are 47 conserved miRNA families and 88 novel miRNAs identified from flower and leaf samples.

The read numbers of the 47 conserved miRNA families varied from 11 (miR5538) to 7,491,182 (miR166), which implies the expression patterns of different miRNA families dramatically differ. In this study, there are 10 highly conserved miRNA families identified, including miR156, miR159, miR166, miR167, miR168, miR319, miR393, miR396, miR403 and miR7972. They expressed in the flower and leaf tissues with at least ten thousand reads, which is in line with the previous study about the correlation between plant evolutionary conservation and expression abundance [39, 40]. Moreover, the highly conserved miRNAs have been proved to be very important for plant growth and development. For instance, miR164 and miR167 affect lateral root development and adventitious rooting in *A. thalinana*, respectively [46]. miR159, miR166 and miR167 regulate the floral organs development [47]. In addition, the read numbers of 9 moderately conserved miRNA families, including miR157, miR171, miR172, miR390, miR394, miR529, miR530, miR894 and miR6300, were more than one thousand in at least one tissue, suggesting that they may have important functions in regulating gene expression, signaling pathways modulation in plant development. Moreover, unlike other miRNAs, the expression level of miR5538 was very low and only 11 reads detected in the flower tissue. However, even though the expression level is not abundant, the lowly conserved miRNAs may participate in the regulation of plant developmental and cellular processes such as flower development and abiotic stress responses [14].

Former studies have shown the novel miRNAs normally expressed at low levels and were hard for detection via the traditional sequencing approaches [3], but in this study there were 88 novel miRNAs identified, via the precursor mapping and the characteristic hairpin structures prediction. To decipher and explore the miRNAs functions in regulating plant development, it is critical to predict the potential target genes of miRNAs. In this study, we employed bioinformatics methods to screen the homologous target sequences of miRNAs. As a result, 2439 genes were predicted as the potential targets of 148 miRNAs. Interestingly, most of the predicted potential targets of novel miRNAs in *O. fragrans* were functional genes, and frequently involved in cellular processes, metabolic processes and response to stimulus. It is worth to mention, some of the predicted target genes were important transcription factors in plant including WRKY and GRAS. It is known that WRKY proteins have important roles when plant encounters and responses to biotic and abiotic stresses [48]. Meanwhile, GRAS proteins possess activities in regulating gene transcription and are important regulators for diverse processes in plant growth and development, including gibberellin signal and phytochrome A signal transduction, radial patterning of root, formation of axillary meristem and gametogenesi [43]. As a perennial, evergreen shrub, *O. fragrans* is known as medicinal plant used in folk medicine. It is usually used as the additive in food, tea and other beverages [17, 18]. There are multiple secondary metabolites (such as flavonoids, anthocyanins) and other important nutrition components isolated from *O. fragrans* [29, 30]. Meanwhile, miRNAs were reported to regulate the biosynthesis of secondary metabolite in various plants [30–34]. For example, several miRNAs, including miR156, miR858 and miR172, played important roles in flavonoid biosynthesis pathway [45].

In this study, the results showed that level of flavonoid in flower and leaf tissue was significantly different. We further found miR858a was down-regulated (Fig. 2) and MYB1 gene was up-regulated in flower tissues of *O. fragrans* (Fig. 5b). Previous studies reported that MYB transcription factors regulate flavonol biosynthesis by interacting with CHI, CHS and FLS genes [35, 48]. Coincidently, our results showed that CHI, CHS and FLS genes were significantly up-regulated in flower tissues of *O. fragrans* (Fig. 5b). Based on our results, we speculate that miR858a might regulate flavonoid metabolism in *O. fragrans* by miR858a → MYB → (CHI, CHS, FLS) → Flavonoids pathway (Fig. 5c), which indicated the potential of medicinal plant derived miRNAs in regulating the secondary metabolite biosynthesis. Also, these studies will facilitate the research on the metabolic engineering to efficiently produce new plant chemical components or combinations of secondary metabolites. However, further investigations are required to demonstrate the detailed mechanisms of miRNAs-mediated regulation of secondary metabolite biosynthesis in *O. fragrans*.

## Conclusions

In this study, high-throughput sequencing technology is used to identify miRNAs in leaf and flower tissues of *O. fragrans*. In summary, a total of 106 conserved miRNAs distributed in 47 families and 88 novel miRNAs were identified. The results of bioinformatics analysis showed that among the conserved and novel miRNAs, 133 miRNAs were differentially expressed in leaves and flowers. Moreover, the potential target genes of miRNAs and the related metabolic pathways were predicted to analyze their functions. In the end, flavonoid content between flower and leaf tissues was measured and potential role of miR858 in regulating flavonoid biosynthesis was explored in *O. fragrans*. Taken together, this study not only provided the genome-wide miRNA expression profiles in the flower and leaf tissue, but also addressed the possible regulatory role of miR858 in flavonoid synthesis in *O. fragrans*, indicating the potential of medicinal plant derived miRNAs in regulating the biosynthesis of secondary metabolite in plants.

## Methods

### Plant materials and total RNAs isolation

The flower and leaf samples were collected from *O. fragrans* ‘Liuye Jingui’ planted on the campus of Huazhong University of Science and Technology in Wuhan, Hubei Province, China. They were identified by Dr. Gang Wu of College of Life Science and Technology in Huazhong University of Science and Technology. All plant materials were owned by Huazhong University of Science and Technology. The collected samples were immediately frozen in liquid nitrogen. Afterwards, total RNAs were isolated via TRIzol (Invitrogen) method, according to the manufacturer’s instructions. The quality and concentration of isolated total RNAs was analyzed via the measurement using NanoDrop spectrophotometer (Thermo Fisher Scientific, Inc.). To check the A260/A280 and A260/A230 values, the purity of the RNA was confirmed. In the end, the RNA samples were stored in − 80 °C freezer for further use.

### Construction of sRNA library and deep sequencing

Small RNA libraries were constructed via the protocol described previously [38, 49]. Briefly, electrophoresis of the total RNAs isolated from *O. fragrans* samples was performed in 15% PAGE gel and the fraction with 18–30 nt in size was collected. Next, the 5′ and 3′ RNA adapters were ligated to the RNA pool via T4 RNA ligase, and the RNAs with 5′ and 3′ adaptors were purified for reverse-transcription and PCR amplification. For each tissue, three biological replicates of cDNA samples of flower and leaf were deeply sequenced using Illumina HiSeq platform, which totally generated ~ 95 and ~ 97 million raw reads, respectively.

The clean reads data was generated after removing the adaptor sequences and filtering the low-quality reads using AdapterRemoval program [50]. The criteria used to define the low-quality reads are listed as below: 1) The reads in which quality scores of more than 15% of the bases are less than 19. 2) The reads in which the percentage of N (N means the bases whose base information cannot be determined) is greater than 10%. 3) The reads without 3′ adaptor sequence. 4) The reads without inserted fragments. 5) The reads containing polyA/T. In addition, rRNA, tRNA, snRNA, snoRNA and the materials containing the poly-A tail were removed, and the left sequences were used for further analysis.

### The conserved and novel miRNAs identification

The length distribution of the clean reads was summarized and the mapping of the clean reads to the *O. fragrans* EST and GSS databases was performed using SOAP [51]. The perfectly mapped sRNAs were obtained for the further study. Meanwhile, the clean reads mapped to rRNAs, tRNAs, snRNAs and snoRNAs in Rfam database [52] were discarded. Furthermore, the remaining sRNAs were mapped with mature miRNA and miRNA precursor in miRBase and less than two mismatches was allowed. The perfectly matched small RNAs were considered as conserved miRNAs. Finally, the secondary structure, the cleavage sites of Dicer complex and the minimum negative folding free energy of the unannotated small RNAs were measured via the RNAfold (http://www.tbi.univie.ac.at/~ivo/RNA/ViennaRNA-1.8.1.tar.gz) and psRobot [42]. The following criteria [53, 54] were set to identify novel miRNAs: 1) The precursor should have a stem-loop structure containing mature miRNA sequence within one arm. 2) Mature miRNA should have fewer than 2 mismatches with its complimentary sequence. 3) The precursor should not contain the mature miRNA in the hairpin structures’ terminal loop. 4) The stem-loop structures for the precursors of novel miRNAs have higher minimum folding free energy index (MFEI).

### Analysis of the differentially expressed miRNAs

To better understand the biological function of the identified miRNAs, it is necessary to analyze the expression levels of miRNAs in leaf and flower tissue and screen the differentially expressed miRNAs. In order to identify the differentially expressed miRNAs between flower and leaf tissues, we set the expression level of the miRNAs in leaf as a control and detected the up- or down-regulated miRNAs in flower. The RPKM (reads per kilobases per million reads) of each miRNA expression in flower and leaf tissues was calculated. The differentially expressed miRNAs were then defined using DEGseq program [55] with abs (log_2_ fold-change) > 1 and adjusted *p*-value <0.01.

### The confirmation of miRNAs expression via quantitative real-time PCR (qRT-PCR)

To indirectly certify the sequencing data quality of small RNAs samples and directly confirm the differential expression patterns of the identified miRNAs in the flower and leaf tissues of *O. fragrans*, we randomly selected some of the miRNAs to perform qRT-PCR experiment. In this study, we chose 16 miRNAs (including 9 conserved miRNAs and 7 novel miRNAs) for qRT-PCR. To generate cDNA, 3μg of each stored total RNA sample from leaf and flower tissues were used for reverse transcription, according to the user manual (#AMRT-0020, GeneCopoeia). The qRT-PCR experiments were independently repeated three times to measure the expression levels of the selected miRNAs in leaf and flower tissues. All the qRT-PCR experiments were performed in the ABI StepOne Plus PCR instrument using miRNA qRT-PCR kit (#AMPR-0200, GeneCopoeia). U6 was used as the internal control in these qRT-PCR experiments and the relative expression levels of the selected miRNAs were calculated using the standard comparative Ct method. For each miRNA, the reactions were triplicate via the sample addition into three wells of the PCR plate. The primer sequences used in this study were listed in the Additional file 8: Table S7.

### Prediction and annotation of the miRNAs target genes

To further illustrate the biological functions of the identified miRNAs, the software psRobot was employed to predict the potential target genes of the identified miRNAs [42]. Then, the functions of the potential target genes were annotated with the Gene Ontology (http://www.Geneontology.org/) and the Kyoto Encyclopedia of Genes and Genomes (http://www.Genome.jp/kegg/).

### Validation of miRNA target by 5′ RLM-RACE

The predicted target gene of miRNA was validated through 5′ RLM-RACE using the First Choice RLM-RACE kit (Ambion, Austin, TX) as previously described (38). The gene-specific primers (Table S7) used for the above experiments were designed according to sequence the predicted target. The cleavage target fragments were cloned into pTA2 vectors (TOYOBO, TAK101) and sequenced using the universal T7 primers.

### Determination of flavonoid content in *O. fragrans*

The flavonoid content of the acetonic extract in *O. fragrans* was measured using a colorimetric method described previously [56]. In brief, 0.25 mL of or (+)-catechin standard solution was mixed with distilled water (1.25 mL) in a test tube and 5% NaNO_2_ solution (75 mL) was then added. 10% of AlCl_3_·6H_2_O (150 mL) was added 5 min later. After standing for another 5 min, 0.5 mL of 1 M NaOH was added. The mixture was diluted to 2.5 mL and the absorbance was measured spectrophotometrically against a blank at 510 nm in comparison with standards similarly with known (+)-catechin concentrations. The results were shown in mean with four replications.

## Supplementary Information

**Additional file 1: Table S1**. Small RNA sequencing analysis for flower and leaf in *O. fragrans*

**Additional file 2: Table S2**: The identified conserved miRNAs and miRNA families in *O. fragrans*.

**Additional file 3: Table S3**: The identified novel miRNAs in *O. fragrans*.

**Additional file 4: Table S4**: The differentially expressed miRNAs between flower and leaf tissues in *O. fragrans*.

**Additional file 5: Table S5**: The predicted miRNA target genes in *O. fragrans*.

**Additional file 6: Fig. S1**: 5′ RLM-RACE analysis of miR858a cleavage site on its target mRNA. Cleavage site of miR858a is shown by the arrow with the frequency of cloned RACE products. The vertical lines indicate matched base pairs

**Additional file 7: Table S6**: The KEGG pathway analysis of target genes in *O. fragrans*.

**Additional file 8: Table S7**. Primers for qRT-PCR validation of differentially expressed miRNAs and mRNAs in *O. fragrans*.

## Data Availability

Sequencing data of *O. fragrans* was deposited in SRA (Sequence Read Archive) Database in NCBI (National Center for Biotechnology Information) with Accession number SRR12277026, SRR12277027, SRR12277028, SRR12277029, SRR12277030 and SRR12277031.

## Abbreviations

*O. fragrans*: *Osmanthus fragrans*
*A. thaliana*: *Arabidopsis thaliana*
miRNA: MicroRNA
qRT-PCR: Quantitative RT-PCR
RISC: RNA-induced silencing complex
GO: Gene ontology
DE-miRNA: Differentially expressed miRNA
RPKM: Reads per kilo bases per million reads
TFs: Transcription factors
