# Supplementary material for: Genome-wide miRNA analysis and integrated network for flavonoid biosynthesis in Osmanthus fragrans
Source: BMC Genomics. 2021 Feb 27;22:141. doi: 10.1186/s12864-021-07439-y (PMC7913170; doi:10.1186/s12864-021-07439-y)
Supplement: Supplementary file 1 — Additional file 1: Table S1. Small RNA sequencing analysis for flower and leaf in O. fragrans [file 12864_2021_7439_MOESM1_ESM.docx]

| **Table S1**: Small RNA sequencing analysis for flower and leaf in *O. fragrans* | | |
| --- | --- | --- |
|  | Flower | Leaf |
| Clean reads | 22,825,464 | 23,131,451 |
| Uniquely-mapped reads | 5,820,493 | 6,127,715 |
| rRNA | 3,841,525 | 4,038,164 |
| tRNA | 1,169,919 | 1,194,904 |
| SnoRNA | 87,307 | 90,627 |
| snRNA | 46,968 | 49,256 |
