# Supplementary figures and images for: Genome-wide miRNA analysis and integrated network for flavonoid biosynthesis in Osmanthus fragrans
Source: BMC Genomics. 2021 Feb 27;22:141. doi: 10.1186/s12864-021-07439-y (PMC7913170; doi:10.1186/s12864-021-07439-y)

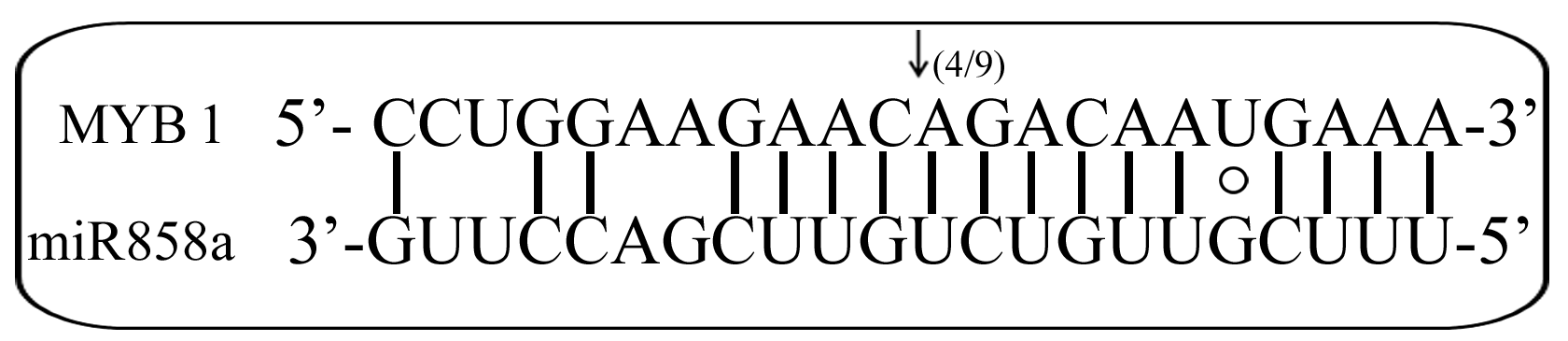

Supplement: Supplementary file 6 — Additional file 6: Fig. S1: 5′ RLM-RACE analysis of miR858a cleavage site on its target mRNA. Cleavage site of miR858a is shown by the arrow with the frequency of cloned RACE products. The vertical lines indicate matched base pairs [file 12864_2021_7439_MOESM6_ESM.tif]
