# Supplementary material for: Genome-wide miRNA analysis and integrated network for flavonoid biosynthesis in Osmanthus fragrans
Source: BMC Genomics. 2021 Feb 27;22:141. doi: 10.1186/s12864-021-07439-y (PMC7913170; doi:10.1186/s12864-021-07439-y)
Supplement: Supplementary file 8 — Additional file 8: Table S7. Primers for qRT-PCR validation of differentially expressed miRNAs and mRNAs in O. fragrans. [file 12864_2021_7439_MOESM8_ESM.docx]

| **Table S7:** Primers for qRT-PCR validation and 5’ RLM-RACE . | | |
| --- | --- | --- |
| miRNAs/mRNA | Forward primer (5’→3’) | Reverse primer (5’→3’) |
| miR858 | TTCGTTGTCTGTTCGACCTT | Universal reverse primer in All-in-One ^TM^ miRNA qRT-PCR Detection Kit (GeneCopoeia) |
| miR167-3p | GATCATGTTCGCAGTTTCACC |  |
| miR398 | TGTGTTCTCAGGTCGCCCCT |  |
| miR4995 | AGGCAGTGGCTTGGTTAAG |  |
| miR157a-3p | GCTCTCTATGCTTCTGTCAT |  |
| miR171 | TGATTGAGCCGCACCAATAT |  |
| miR319 | AGAGCTTCCTTCAGTCCACT |  |
| miR390a | UAAAGCUCAGGAGGGAUAGC |  |
| Novel_6 | AAGAGGCATCTAAGAGGGGA |  |
| Novel_80 | TTGGCTGGGCTTATGCTTG |  |
| Novel_35 | CCTGAGTTGTGCCAAATGAA |  |
| Novel_62 | TCGGACCAGGCTTCATTC |  |
| CHS | ATTACTGCGGTTACATTCCG | TCCCATACTCGCTTAGCACA |
| CHI | TCCTCCATTCTATTCACTCA | AACGATTATTTGCTGCTAT |
| F3H | TGTTTGGCTCGTGAGTTCTT | CCGCTTTAGCCCAAGTGTTA |
| MYB1 | AAGTGGGGAAACAGGTGGTC | TCCATCAATCGACGGTGGAG |
| MYB1 inner | GCTTGTGCTTGCTTGATCGT | |
| MYB1 outer | CCATCAATCGACGGTGGAGA | |
